# Establishing views of traditional healers and biomedical practitioners on collaboration in mental health care in Zanzibar: a qualitative pilot study

**DOI:** 10.1186/s13033-020-0336-1

**Published:** 2020-01-09

**Authors:** Lindsay Solera-Deuchar, Mahmoud I. Mussa, Suleiman A. Ali, Haji J. Haji, Peter McGovern

**Affiliations:** 1 South London & Maudsley NHS Trust, Bethlem Royal Hospital, Monks Orchard Road, Beckenham, BR3 3BX UK; 2 Commission for National Coordination and Drug Control, Zanzibar, Second Presidents’ Office, P.O.Box 1855, Zanzibar, Tanzania; 3 Ministry of Health, PO Box 236, Mnazi Mmoja, Zanzibar, Tanzania; 4 Zanzibar Research Institute, Ministry of Health, PO Box 236, Mnazi Mmoja, Zanzibar, Tanzania; 5 Modum Bad, Badeveien 287, 3370 Vikersund, Norway

**Keywords:** Traditional healers, Collaboration, Mental health, Zanzibar

## Abstract

**Background:**

This qualitative pilot study aimed to establish views of traditional and biomedical practitioners towards collaboration between the two sectors on the treatment of people with mental illness in Zanzibar, Tanzania.

**Methods:**

Six traditional healers (known as “waganga” in Swahili) and six nurses working in government secondary mental health services were invited to participate in a series of focus group discussions (FGDs). Two sets of FGDs took place approximately seven weeks apart. In each set, FGDs were conducted with traditional healers only, nurses only, and finally nurses and traditional healers together. FGDs were conducted in Swahili, audio-recorded and then translated to English by an independent translator and coded thematically using NVivo software.

**Results:**

All participants expressed that they were in favour of collaboration between traditional and biomedical practitioners on mental healthcare. Opinions varied regarding what form this collaboration should take. For many nurses and healers, there was acknowledgement of the role of the other group in providing treatment for people with mental illness, with support for the idea of bi-directional referrals between the two sectors. For some nurses, the value of collaboration would be purely in the education of traditional healers in the recognition of mental illness, with subsequent referral to biomedical services. For some traditional healers, the idea of collaboration seemed to appeal in part because of a perceived opportunity to learn additional skills from biomedical practitioners. Both categories of participant expressed a belief that patients possessed by a *jinn* (a spirit) or those that had been bewitched needed treatment by traditional healers. On the other hand, those with what participants considered to be “mental illness” needed treatment at the hospital clinic. However, some nurses felt that that traditional healers might be able to provide helpful treatment for mental illness, as well as those suspected to be affected by *jinn* or witchcraft. There was agreement on the need to establish clear referral pathways between the two service providers. The creation of an office for traditional healers at the hospital was an area where there was disagreement among participants.

**Conclusions:**

We conclude that there is a positive view of collaboration among traditional healers and nurses who participated, and a willingness to work towards actual collaboration. The results suggest that views vary as to what form this collaboration should take, with opinions differing between nurses, as well as between traditional healers. Additional work is needed in order to further explore the nature of potential collaboration and extend the research to the wider population of traditional and biomedical practitioners in Zanzibar, to include primary health care workers.

## Background

**Fig. 1:**
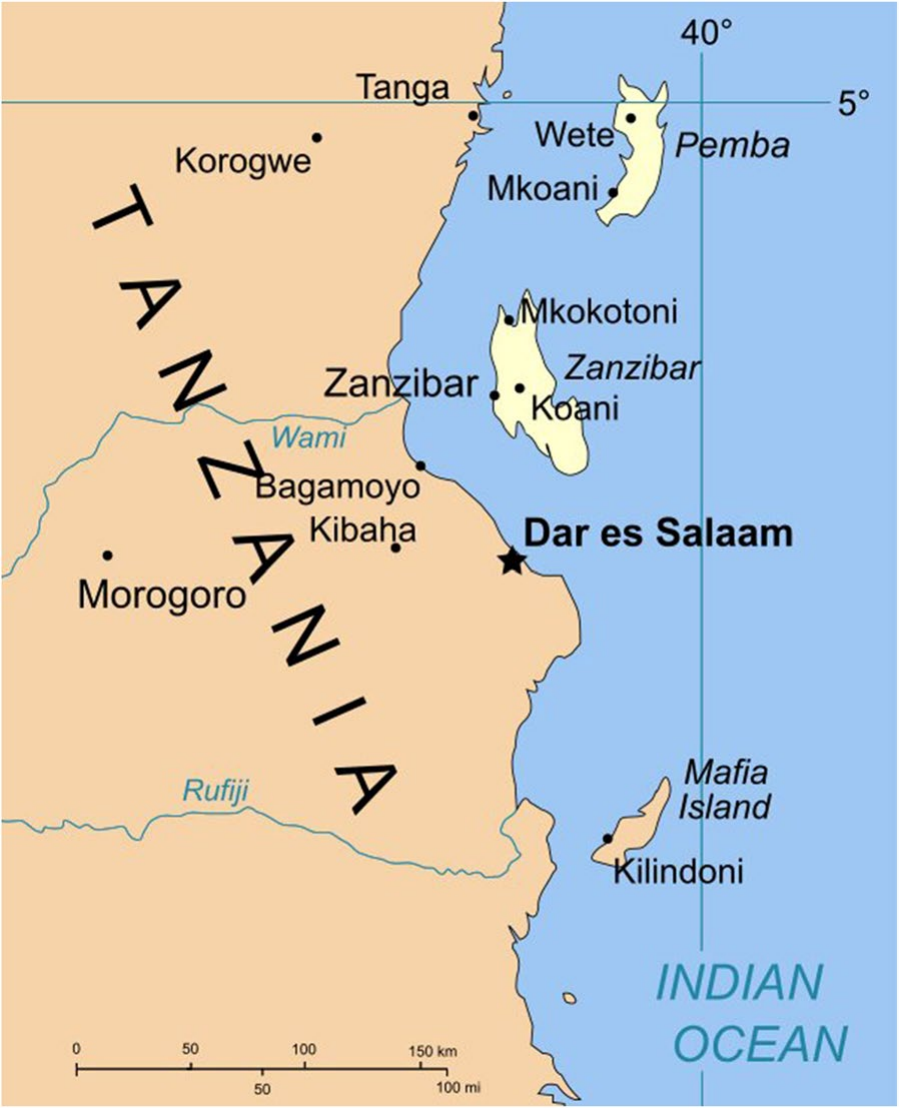
Map of Zanzibar (from Wikimedia Commons, free media repository)

Zanzibar is an archipelago off the coast of East Africa, a semi-autonomous region of Tanzania (Fig. 1). The two main islands, Unguja and Pemba have a population of 1.3 million according to the 2012 population and housing census [1]. For historical reasons, Zanzibar maintains its own president and semi-autonomous government, including a Ministry of Health and Social Welfare.

Traditional healers or *waganga wa kienyeji* are highly relevant to the care of people with mental illness in Zanzibar, where 99% of the population is Muslim, and there is a strong belief in the practice of witchcraft, or *uchawi*, involving malicious spirits known as *jinn.* The Qur’an describes *jinn* as invisible beings created by Allah, and according to local beliefs, people are susceptible to possession by *jinn* during transitional periods of their lives [2]. Although possession by *jinn* is seen as a usual part of Swahili culture [3], psychiatric symptoms are commonly attributed to *jinn* [4], and patients often seek help from traditional healers for mental illness [5].

It has been estimated that there are at least 800 traditional healers working across Unguja and Pemba [6]. More widely, in Sub-Saharan Africa the number of traditional healers is approximately one hundred times the number of conventional medicine practitioners [7].

Traditional medicine in Zanzibar has a broad definition, including herbal medicine, Qur’an medicine, scarification, spiritual matters and divination [6]. In a study in Zanzibar, a number of patients interviewed who had discontinued their attendance at biomedical clinics stated that they had done so because they had decided to switch to traditional medicine instead [6]. Patients reported attending traditional healers more readily than biomedical clinics for a number of reasons, including accessibility, affordability and preference for the treatment provided by traditional healers.

Reviews of mental health services in Zanzibar show that the majority of patients present to traditional healers before visiting mainstream practitioners and the Zanzibari ministry report that the use of their services is increasing in line with the population, due to scarcities of medication in the public sector [8]. In 2008 the Ministry of Health released the *Zanzibar Traditional and Alternative Medicine Policy*, recognising the potential benefits of regulating the practices of traditional healers and enabling a closer relationship with mainstream practitioners [9]. This includes reducing the risk of traditional healers engaging in harmful practices, as well as recognising them as a resource rather than a threat or danger. However, the role of traditional and alternative medicine in mental health care is not addressed in this policy, and it is an area that has not been previously explored in Zanzibar.

Collaboration between biomedical and traditional services could allow for the transfer of skills and knowledge, and indeed the World Health Organization (WHO) has repeatedly stressed the importance of collaboration between the two sectors [10]. A study in Zanzibar in 2012 that looked at the possibility of collaboration in the general health sector demonstrated that traditional healers are prepared to collaborate with doctors, and doctors acknowledged that patients’ mental health could benefit through the holistic approach from traditional healers, who often provide counselling [6]. This holistic approach has been shown to be particularly appreciated by patients with mental health problems [7]. Traditional healers could also offer a more culturally-acceptable, less stigmatised and more varied approach to treatment which is consistent with patient beliefs about the causes of mental illness. However, until recently in Zanzibar, there has been no system of referral between biomedical practitioners and traditional healers for mental health problems, and there hasn’t been any formal discussion about collaboration in this field.

A literature search demonstrates a number of studies conducted in other parts of sub-Saharan Africa that have brought together traditional healers and biomedical practitioners for discussion of collaboration on mental health care, although none as yet in Zanzibar. Two studies in Kenya found that the majority of practitioners (including traditional healers, faith healers and formal health care workers) were willing to collaborate, and showed recognition of the benefits of collaboration [11, 12]. A study in South Africa found that traditional healers and biomedical practitioners felt that cooperation could lead to the provision of more culturally appropriate treatment for patients [13]. Some traditional healers reported already referring patients to biomedical practitioners. Another study in Kenya involving focus group discussions with a large number of traditional healers found that traditional healers were able to identify some mental illnesses, in particular psychoses, and were willing to cooperate with biomedical practitioners and refer patients to them [14].

A common theme across the above studies was that traditional healers respected and valued biomedical practitioners but felt disregarded by the biomedical profession. Traditional healers often report sending referrals to biomedical practitioners, but not receiving any in return. The importance of recognising the value of traditional healers is emphasised in The *Joint United Nations Programme on HIV/AIDS* (UNAIDS) recommendations for collaborating with traditional healers on HIV, which is relevant to potential collaboration on mental health in Zanzibar [15]. Their recommendations include acknowledging the status and power of traditional healers, and recognising that traditional medicine can be more holistic. They suggest discussing openly differences, and what the strengths and failures of each group.

The aim of this pilot study, therefore, was to open dialogue between traditional healers and biomedical practitioners in Zanzibar on the subject of collaboration in mental health care. A study of this kind is yet to be done in Zanzibar, an island which has a distinct history and culture from mainland Tanzania. We aimed to establish opinions on collaboration, and explore the possibility of biomedical practitioners and traditional healers working together on the care of people with mental illness in Zanzibar.

## Methods

### Participants and setting

Six traditional healers practising in the Kivunge Hospital catchment area (North District) and six nurses working in secondary mental health services in Unguja were contacted and invited to participate in the study. Participants were chosen using convenience sampling. Staff from secondary care were included rather than primary care because currently it is largely in secondary care that patients with mental illness are accessing treatment. Participants were reimbursed for transport costs and given lunch and refreshments. No other payments were made to participants.

Six traditional healers and six nurses attended all focus group discussions. Four traditional healers were registered with the Zanzibar Traditional Medicine Unit, whereas two were unregistered. Two nurses worked at the Kivunge cottage hospital psychiatric clinic, two at the Makunduchi cottage hospital psychiatric clinic (South district), and two at Kidongo Chekundu psychiatric hospital in the island's capital, Zanzibar City. All participants were male.

The focus groups took place at Kivunge Cottage Hospital, which was easily accessible by all participants.

Participants gave their written consent after reading an information sheet in Swahili explaining the study, and being given the opportunity to ask any questions. An English version of the consent form is included as Additional file 1.

At the beginning of the first discussions, participants were asked to explain what kinds of treatment they use in their clinics. Traditional healers reported using herbal medicines, the Qur’an and holy water. Nurses reported using medication, counselling and psychoeducation.

### Design and procedure

Two sets of focus group discussions were held between May and July 2017, seven weeks apart. At each set, three separate focus groups were held, the first with traditional healers only, the second with nurses only, and the third with traditional healers and nurses combined. The participants were separated in this way initially as the authors felt that they might feel more free to express more negative views when in the company of their peers only. The two sets of focus group discussions were held seven weeks apart in order to explore whether the focus group discussion in itself might lead to changes in opinions towards collaboration and even a change in practice.

The discussions were conducted in Swahili, chaired by MM (a native Swahili speaker) and assisted by LSD (second language Swahili speaker). MM led the majority of the discussion, with LSD contributing some additional questions. MM ensured that each member of the group had the opportunity to give a response to each question if they wanted to. Each of the six individual focus groups lasted between 30 and 60 min.

### Instruments

The initial focus group discussions were based around a topic guide, which consisted of a list of pre-determined questions read aloud by MM with additional questions sometimes arising from participants’ responses. The questions aimed to stimulate discussion on collaboration between the two groups, first exploring the role of each group in the treatment of mental illness, followed by the benefits and challenges of collaboration.

The subsequent focus group discussions (seven weeks later) aimed to explore any changes in attitudes towards collaboration as a result of the first set of focus groups, as well as the more practical questions of collaborating. The pre-determined questions were designed to include exploration of themes that arose in the first set of focus groups, such as the question of an office for traditional healers at the hospital, and the exchange of knowledge between the two groups. The topic guides are included as Additional file 2.

### Data analysis

The audio recordings of the conversations were transcribed and translated into English by professional translators at the State University of Zanzibar, and a qualitative analysis was conducted. The data was inputted into NVivo data analysis software, and line-by-line coding used to categorise comments. Open coding was used, with no pre-determined codes. Emerging themes were then identified and agreed upon by a team of researchers. Contributions of traditional healers were compared with those of nurses within individual themes, and comments made during traditional healer-only or nurse-only focus groups were compared with mixed focus groups. The team of researchers included one non-clinician with no involvement biomedical mental health services.

## Results

The seven main themes arising during the discussions are summarised below, with examples of statements made by participants during the discussions. It was found that there was no notable difference between opinions expressed in traditional-healer-only or nurse-only focus groups versus mixed focus groups, and so the results are summarised together.

There was also no observable shift in opinions between the first and the second set of focus groups, although several participants said that they felt more positive about collaboration as a result of the first discussions. However, all participants had expressed positive views in the initial set of discussions. The results of the first and second sets of focus groups discussions are therefore summarised together.

### Beliefs about the causes of mental illness

All participants agreed that mental illness is a problem in Zanzibar. Both traditional healers and nurses expressed a clear belief that there was a difference between mental illness, as caused by God, and problems caused by *jinn* or evil spirits. Both parties felt that mental illness should be treated at the hospital, whereas problems relating to *jinn* should be treated by the traditional healer.

*“I think there are mental health patients and people with* jinn*, so mental health patients should be taken to hospitals and people with* jinn *should be brought to us. Because* jinn *is caused by witchcraft, but mental illness arises from God’s will.”* (Traditional healer)

*“And I think it would be wise if they can say that this is not witchcraft, let’s send our patient there [to the hospital]. The same goes for us, when we find some indication of witchcraft we’ll be able to send patients to them.”* (Nurse)

*“We are all believers and we all believe in the existence of witchcraft. If you do not believe in witchcraft then you’re not among us.”* (Nurse)

However, there was no agreement on how one would decide whether a patient had a *jinn* problem, or a mental health problem. Nurses expressed feeling unaware of how traditional healers make their diagnoses, and vice versa.

*“Me as a health care worker, I would like to know those professional techniques they use to claim that he/she is bewitched because it happens as soon as a person enters their room; when a traditional healer simply sees a patient, he already knows that they are bewitched. So I would like to know what sign they use to diagnose the mental health patient.”* (Nurse)

*“And I would like to know what procedures they have in hospital and tests indicating that these are hospital diseases.”* (Traditional healer)

### Roles of traditional healers and health care workers in treating mental illness

Traditional healers recognised the role of health care workers in treating mental illness.

*“In reality, mental health patients depend on our traditional treatment a lot, but we surely depend on health care workers because they have scientific medical tests which are very valid, but we as traditional healers have to practise by reading stars which takes some time before finding the problem. But if a patient goes to a doctor, they find the problem within five minutes. So we are often chosen by patients, but we often send patients to doctors.”* (Traditional healer)

Several nurses recognised the role of traditional healers in treating mental illness, acknowledging that many individuals with mental health problems first access traditional healers.

*“But we just experience that many patients start with treatment from the traditional healers, and if they are not improving then they come to us.”* (Nurse)

*“On my side, I feel motivated to work with them, because traditional healers have a key role in addressing mental health problems. So I’m motivated and I’ll share with them.”* (Nurse)

However, one nurse felt that traditional healers’ contribution was not important.

*“The mental illness is well treated at the hospitals. I think the traditional healers have a very small contribution on this.”* (Nurse)

There was recognition of the validity of different perspectives on treating patients with mental health problems.

*“I am not going to argue because each side is right on its part.”* (Nurse)

*“There will be a lot of perspectives, and each health care worker has his/her own perspective.”* (Healer)

### Opinions on collaboration between the traditional and biomedical sectors on mental health

All participants made comments suggesting that there is a need for collaboration between traditional healers and health care workers on mental health. A sample of these are included below:

*“Our goals and desires of collaboration are to be together; for a long time health care workers and traditional healers worked separately. So this collaboration will bring us to know each other and work together as a team.”* (Traditional healer)

*“If we work together, it will reduce the time for the patient to recover*—*he/she will recover more quickly through collaboration. If we stick together, then the patient will recover in a short time.”* (Traditional healer.)

*“My opinion is to see our relationship stabilise even more and work as a family, if our main goal is to help these mental health patients.”* (Nurse)

*“There are many advantages…it saves the costs for the people, because when we cooperate in treating the patient quickly we are definitely reducing costs.”* (Nurse)

No participants expressed being against the idea of collaboration, although as other themes reveal, opinions differed regarding what form collaboration should take.

### Exchange of knowledge between traditional healers and nurses working in mental health

A theme that was raised by participants in the first set of discussions and subsequently explored further was the exchange of knowledge between traditional healers and nurses. There was strong support for education of traditional healers by health care workers on mental illness from both traditional healers and nurses. Nurses generally felt that education of traditional healers was important for the purposes of referrals to hospital, whereas traditional healers seemed to want to gain knowledge of diagnosis and treatment for use in their own clinics.

*“In my opinion many patients who come to the hospital are few compared to those who go to the traditional healers clinics, therefore they have to be educated so that the same large number of patients they receive will be easier to bring to us. Hence without giving them education, things will remain the same.”* (Nurse)

*“What I want to learn from the health care workers is about scientific diagnosis of the mental health patient; how do they determine a mental health patient by using scientific methods of examining a patient?”* (Traditional healer)

Several traditional healers expressed a wish to learn about medication used by health care workers to sedate patients with mental illness.

*“I would like to learn from health care workers about the medication they use to make a mental health patient calm.”* (Traditional healer)

There was also support from nurses for receiving knowledge from traditional healers, but this idea was not commented on by traditional healers. Nurses indicated that they were curious to know what techniques traditional healers use for diagnosis of witchcraft or *jinn* as well as how they treat these patients.

*“As a health care worker I would like to learn more about the techniques they use in identifying symptoms of mental illness. Techniques such as those traditional practices they use to identify mental health patients.”* (Nurse)

“*I would like to learn about how to determine that a mental health patient may be someone who has been bewitched and secondly about what medicines traditional healers use as well as what verses in Qur’an they recite to make a patient calm.”* (Nurse.)

### Opinions about bi-directional referral of patients between traditional healers and the hospital

Several traditional healers stated that they felt patients with mental illness should first attend the hospital, and then the traditional healer’s clinic if there was no improvement.

*“The patient should be treated by health care workers first but if it doesn’t work then bring the patient to us*.” (Traditional healer)

*“The first priority for any mental health patients is the hospital.”* (Traditional healer)

*“My opinion is that any mental health patient should be taken to the hospital, he/she will scientifically be tested as usual and if there’s no sign of illness then there’s need for collaboration”.* (Traditional healer)

Nurses felt strongly about the need for patients with mental illness to be seen at the hospital.

*“Yes there are some patients who should be treated at the hospital, a mental health patient who is paranoid, the patients who are threatening to commit suicide, these should be brought to the hospital for protection and to be treated with drugs that will help them to think better.”* (Nurse)

*“Every mental health patient needs to be seen at the hospital. If he/she has some symptoms of mental illness, it is possible for all of us to see it as a small matter today, but tomorrow it will be bigger. So even when the treatment is at home, he/she must be taken to hospital for a check*-*up, so that the hospital can do a scientific investigation. For example, there are social workers who visit patients at home. I think it’s important for every patient to get a hospital registration, and then we’ll know how many mental health patients we have in Zanzibar.”* (Nurse)

Nurses also recognised, however, the value of referring patients to traditional healers.

*“Therefore the collaboration is still needed and to send a patient to either health care workers or a traditional healer doesn’t mean that you devalue yourself; it is just to help that patient. No, it’s just that I’ve reached my limit, so I must send the patient to them, and if they can’t help, they’ll send them back to me.”* (Nurse)

*“And I think it would be wise if they can tell us that this is not witchcraft, and send their patient to us. The same goes for us, when we find some indication of witchcraft we’ll be able to send to them.”* (Nurse)

One nurse expressed concerns about referring patients to traditional healers, however.

*“My faith does not allow sending a patient to them because I don’t believe that they can cure him/her though I’m not familiar with their services.”* (Nurse)

Another nurse raised that there might be economical barriers to referrals to hospital from traditional healers.

*“I think there’s a problem because hospital staff receive monthly wages even if they don’t treat any mental health patients, but for traditional healers the patient is their salary, so it could be difficult to send a patient to the hospital, as they would fear losing income and this could create resentment between us.”* (Nurse)

Numerous suggestions were made by traditional healers regarding ways to facilitate referrals and collaboration with the hospital, with the theme of increasing respect for traditional healers at the hospital. Both traditional healers and nurses independently suggested the need for forms for referrals between traditional healers and the hospital.

*My contribution is that in order to create a strong relationship, it is better to have referral forms which will be helpful to transfer patients between health care workers and traditional healers, specifically for mental health patients. Therefore, create us those forms for good collaboration.* (Traditional healer)

*“Maybe we should create a form for them containing all the necessary information, like names and other information. Then if they want to transfer a patient they have to follow the procedure on the form and explain the problem in detail with a short history about the patient.”* (Nurse)

A suggestion was made that identification badges would ease access to the hospital for traditional healers, as well as identifying specific clinicians that traditional healers should approach with patients with suspected mental health problems.

*“With those referral papers, at the hospital you will be permitted to pass; you’ll show them the identification card and automatically they’ll recognise you as traditional healers and know that you’re seeking collaboration.”* (Traditional healer.)

*“If they see traditional healers at the hospital with a patient, even if there is a long queue you’ll go directly with your referral and the patient will get treatment; so if we do so it will be of great help.”* (Traditional healer.)

*“I would like Doctor X to be allocated to us, the doctor to whom we have to entrust our patient or any doctor who is closest to him”* (Traditional healer)

### Opinions about the simultaneous use of traditional and biomedical treatments

Participants discussed the benefits and risks of patients using both traditional and biomedical treatment methods simultaneously.

There was much concern, particularly from nurses, about the physical health risks of combining herbal remedies and hospital medicines. It was felt that this risk would be reduced through collaboration.

*“We will tell him/her not to mix with other medication. One problem is mixing the medication, and we do not know which medication is good for him/her, but if there’s cooperation, we may know what medicine is working for him/her.”* (Nurse)

However, both nurses and healers recognised that there were kinds of treatments from both sectors that could be combined.

*“I am confident that we can use both treatments at the same time. If on one side you’re using medication, and on the other you’re using treatments that aren’t medicine, such as conversations, if the patient needs counselling, psychological therapies, or maybe for those who can practise religious prayer they can do it while the patient continues using his/her hospital medicines.”* (Nurse)

*“If we have been called here to the hospital to see a mental health patient, we should pray to God but each in his/her own way without giving a patient any tablets.”* (Traditional healer)

There was recognition from nurses and traditional healers that a patient’s belief about their illness should influence the treatment they receive.

*“The thing that cures is a person’s faith; this can make him/her recover, so we want to look at what makes him/her recover, so that they are able to work.”* (Nurse)

*“A hospital patient might come to us first or to the hospital first and when he comes he/she will say I’ve been to the hospital but I’m still not well and if his/her belief is in traditional treatment then he/she can get well.”* (Traditional healer)

### Opinions about a traditional healers’ office at the cottage hospital

In the first set of focus groups, the idea of setting up a traditional healers’ office or clinic at the hospital was raised. This was explored further in the second set of discussions.

All traditional healers and the majority of nurses were in favour of the idea.

*“I think there must be a special traditional clinic in the hospital then we can have a chance to treat mental health patients as well as creating good partnership, do you agree?”* (Healer)

*“So however it happens, we should reach a place where we can put an office, to say, are you happy to start treatment, or according to how you are feeling, do you think it would be better to go to the other side, and we can prepare a referral.”* (Nurse)

Two out of six nurses were against the idea, largely because of disturbance to other patients from the mode of treatment that traditional healers use. However, there was also mention of concern that patients would be encouraged to use traditional instead of biomedical treatment.

*“I disagree with the existence of a special ward for traditional healers at hospital. Their service will cause disturbance toward other patient because their mode of treatment may include dancing to drums, practising divination or offering a prayer for the patient which obviously sounds loud at the hospital. It is not easy for them to perform their treatment here due to the unsupportive environment. It would be OK if it was only conversation, but we know their ways of working are different. And it’s possible that we would witness a number of patients going to that special ward for traditional treatment instead of hospital.”* (Nurse)

One nurse expressed awareness of the challenges that would be faced, having heard about similar initiatives at hospitals in mainland Tanzania.

*“I am still insisting that having an office or special ward is possible but it needs intensive preparation to be an office. Setting up an office without preparation will cause chaos because health care staff will come out with different perspectives inhibiting the process of setting up a traditional clinic in the hospital. Let’s learn from other hospitals like Muhimbili national hospital in Dar es Salaam—they have a traditional clinic, Tanga hospitals also have it. I am sure that there are more advantages than disadvantages. They were ready to accept challenges and this is why they succeeded. Then we have to learn from them if we really want it.”* (Nurse)

## Discussion

This is the first qualitative focus group study to explore the views of traditional healers and health care professionals on collaboration in mental health care in Zanzibar.

Participants had the opportunity to express their opinions both in a group with their peers, as well as with participants of the other category. This was designed to increase the likelihood of participants feeling comfortable to voice their opinions, as well providing an opportunity for the two groups to discuss the logistics of collaboration in more detail. In reality there was no notable difference in the opinions expressed when amongst peers only versus in the mixed groups, which would suggest that discussions were open and honest. However, it is not possible to exclude some desirability bias, particularly in relation to traditional healers’ views, given that the facilitators were representatives of biomedical mental health services.

The study findings concur with other studies of this kind in Sub-Saharan Africa, which have found positive opinions on the idea of collaboration between the two sectors on mental health care. The results demonstrate that both nurses and traditional healers recognise the importance of collaborating on the care of people with mental illness in Zanzibar. Both groups appreciated the contribution of the other group in treating patients with mental illness, and expressed enthusiasm for collaboration.

The discussion suggested differences of opinion in what form this collaboration should take, and what its purpose should be. In general, comments from nurses suggested that the main purpose of collaboration would be to increase access to mental health services, via traditional healer referrals. However, a few nurses expressed an appreciation that traditional medicine could be of benefit where biomedical services reach their limit. One nurse was clearly against referrals from biomedical services to traditional healers, whereas the remainder were open to the idea. The discussion that arose regarding an exchange of knowledge between the two groups suggest a possibility that the motivation for collaboration for some might come from a desire to learn new techniques that could be used in participants’ own practice. It is unclear from these conversations why this might be, however it is worth bearing in mind that both groups of participants could in theory increase their income by expanding their practice (in the case of nurses through private work). As one participant raised, the issue of income gain and loss through collaboration is relevant particularly to traditional healers, and warrants further exploration. However, several traditional healers made comments about collaboration that focused on the benefit to patients.

The conversation progressed beyond views on collaboration to more detailed discussions on the practicalities of this, including mutual exchange of knowledge, referral pathways and increased recognition of traditional healers at the hospitals. There was recognition of the value of both kinds of treatment for patients with mental illness, even simultaneously in some cases, provided herbal treatments and medication are not used together.

Several traditional healers and nurses expressed the view that patients should be seen at the hospital first, and then by the traditional healer if there was no improvement. However, as one nurse expressed, and as previous research corroborates [6], the reality is that patients tend to consult the traditional healer first. The idea that traditional healers could act as first responders, with the education and knowledge to refer patients to biomedical mental health care services if needed is one that would merit further exploration.

The discussions raised an interesting distinction between mental illness believed to be caused by God, treatable by health care professionals, and mental illness caused by witchcraft or *jinn,* requiring treatment from a traditional healer. This was mentioned by several participants, both nurses and traditional healers, although it was unclear how the distinction was made. This needs further exploration, as it is possible that such a clear distinction could lead to patients being restricted to one service or the other, when in fact they might benefit from both. However, nurses’ belief in the need for traditional medicine for some people with mental illness may explain their respect for traditional healers, and this could increase the likelihood of true two-way collaboration. A focus group study of this kind in Kenya found that traditional healers felt demeaned by health care workers [16], and a qualitative study in South Africa found that health care workers were less interested in a collaborative arrangement than their traditional healer counterparts [17]. It would appear that the interest in collaboration in Zanzibar is more bi-directional than it is in these other communities, which could be related to nurses’ belief in the role of traditional healers in treating certain patients with mental illness in Zanzibar. Indeed in a study exploring collaboration between the two sectors in the general health care sector more generally found similar results, with health care workers valuing and respecting traditional healers as well as vice versa [6].

One limitation of the study is that the participants were exclusively male. This reflects the male predominance of both traditional healers and the nurses working in mental health in the studied catchment areas. However, a recent initiative being introduced by Health Improvement Project Zanzibar (HIPZ), The Friendship Bench [18], involves the training of largely female community health volunteers to deliver talking therapy in the community. This may help to redress the balance in gender of those providing support to those with mental distress or illness, and it would be of value to bring together traditional healers and these community health volunteers for a discussion on collaboration.

Another limitation of the study is that representatives of primary care were not involved, as there is currently very little treatment of mental illness in primary care settings. In practice, collaboration between the traditional sector and the biomedical sector would need to occur at primary care level, given the large ratio of traditional healers to secondary care mental health care professionals. The Ministry of Health and HIPZ are currently working on training of primary health care staff and supply of medication to selected primary health care units. It would be beneficial therefore to conduct a similar larger study exploring the views of primary care health care workers, and their traditional healer counterparts in a sample of communities in Zanzibar.

Finally, the study only included traditional healers known as *waganga wa kienyeji*, who work with herbal remedies as well as readings from the Qu’ran. There are however a large number of religious healers in Zanzibar, whose work may also be relevant in the treatment of people mental illness. Future studies should include these healers.

## Conclusion

In summary, this study suggests that both traditional healers and secondary health care workers in the biomedical mental health sector in rural Zanzibar recognise the value of collaboration, and are optimistic about the possibility of working together. Although views varied regarding the form that this collaboration should take, the results indicated that there is potential to develop the dialogue between traditional healers and biomedical practitioners further. Collaboration is vital in order to improve access to biomedical mental health services for those who need it, as well as to ensure that treatment which is in keeping with patients’ beliefs and cultural norms is available. There was wide support in both groups for exchange of knowledge and two-way referrals. Traditional healers were in favour of establishing an office for traditional healers at the hospital, which was supported by some but not all nurses. A further study is needed to confirm that the views of primary health care workers are similar.

In addition, it would be valuable to explore in more detail the ways in which collaboration between the sectors can facilitate access to safe and culturally relevant treatment for mental illness, whilst ensuring that those requiring biomedical mental health services are able to access it. Further clarity is also needed regarding the perceived distinction between mental illness caused by God, and that caused by *jinn,* in order to ensure that both traditional healers and health care workers are in agreement on who requires which kind of treatment, and who might benefit from both.

Since this study, HIPZ has continued to meet regularly with traditional healers and a formal referral pathway from traditional healers to the biomedical mental health services has been established, with the approval of the traditional healers’ union as well as the Ministry of Health in Zanzibar.

## Supplementary information

**Additional file 1.** Consent form (English version).

**Additional file 2.** Topic guides.

## Data Availability

The datasets generated during and/or analysed during the current study are available from the corresponding author on reasonable request.

## Abbreviations

FGDs: focus group discussions
HIPZ: Health Improvement Project Zanzibar
WHO: World Health Organisation
mhGAP: Mental Health Gap Action Programme
PHCUs: Primary Health Care Units
UNAIDS: The Joint United Nations Programme on HIV/AIDS
