# Supplementary material for: Establishing views of traditional healers and biomedical practitioners on collaboration in mental health care in Zanzibar: a qualitative pilot study
Source: Int J Ment Health Syst. 2020 Jan 9;14:1. doi: 10.1186/s13033-020-0336-1 (PMC6950788; doi:10.1186/s13033-020-0336-1)
Supplement: Supplementary file 1 — Additional file 1. Consent form (English version). [file 13033_2020_336_MOESM1_ESM.docx]

**Research project: attitudes of traditional healers and biomedical practitioners towards collaboration with each other on mental health care - informed consent form**

**Project lead: Dr Lindsay Solera-Deuchar**

You are being invited to take part in a research study. It is your choice whether or not you take part.

Before you decide whether to take part, it is important for you to understand why the project is being carried out and what the study involves. If something is not clear or you would like more information, please ask.

**Why are we doing this study?**

We would like to understand more about the roles of traditional healers and nurses and doctors in looking after people mental health problems. We would like to bring these groups of people together to discuss their strengths and weaknesses in this area, and discuss whether they feel they can work together, in order to provide a better service for people with mental health problems. We would like to tell other hospitals and clinics about what we find, by publishing what we find in a medical journal. We would also tell people who have given us money to do the research.

**What are we asking you to do?**

If you agree to take part, we will ask you to be involved in some group discussions on the topic explained above. The discussions will take place on two days, and on each day, traditional healers will discuss together separately, as will the nurses, and then they will come together to discuss further.

**Who will listen to the discussions, or hear about what was discussed?**

Two researchers will facilitate the discussions, and will record the conversations, which will then be written down. Only the team of researchers will have access to the recording and written version, and the research team will combine participants’ answers before the results are shared. This means that nobody reading the report will know what you personally said.

**What are the risks and benefits of taking part?**

There is very little risk to taking part. There is a possibility you might find some of the discussion upsetting, for example, if somebody disagrees with your opinion. If you do feel upset at any time, you can stop taking part. The benefits of taking part will include improving the relationship between traditional healers and nurses working in mental health, and we hope an agreement might be reached on how the two groups can work together.

**Who is leading the project?**

Dr Lindsay Solera-Deuchar, a volunteer doctor with Health improvement Project Zanizbar, who support the running of Kivunge and Makunduchi Hospitals, will be leading the project with the help of Mr Haji Juma, from the Zanzibar Council of Traditional Medicine, and Mr Suleiman Ali, coordinator of the mental health programme in Zanzibar.

**Do you have to take part?**

You do not have to take part. If you decide not to take part, there will be no bad effects on your work. If you change your mind after the project is completed, you can withdraw your answers from the project up to one month after the completion of the project by contacting the researchers.

**If you would like to talk to the researchers you can contact:**

**Dr Lindsay Solera-Deuchar (project lead) – *[telephone number]***

**Mr Suleiman Ali (co-researcher) - *[telephone number]***

**Mr Juma Haji (co-researcher) - *[telephone number]***

**If you would like to raise complaints or concerns about the study to an independent person, you can contact:**

**Dr Marajani (Head of ZAMREC) – *[telephone number]***

I ________________________________ (name of participant) have been read this information about this study, and all my questions have been answered. I agree to participate in this study with my own consent.

_______________________________ Date:________________

Signature of the participant

_______________________________ Date:________________

Signature of the investigator
