# Supplementary material for: Establishing views of traditional healers and biomedical practitioners on collaboration in mental health care in Zanzibar: a qualitative pilot study
Source: Int J Ment Health Syst. 2020 Jan 9;14:1. doi: 10.1186/s13033-020-0336-1 (PMC6950788; doi:10.1186/s13033-020-0336-1)
Supplement: Supplementary file 2 — Additional file 2. Topic guides. [file 13033_2020_336_MOESM2_ESM.docx]

**Topic guides**

First set of focus group discussions

*Questions for separate focus group discussion for nurses OR traditional healers*

- What kinds of therapies do you use in your clinics?
- What kinds of illness do you treat in your clinic?
- In your view, is mental illness in Zanzibar a problem?
- At the moment, who treats people with mental illness in Zanzibar?
- What is your role in the treatment of people with mental illness in Zanzibar?
- Can you give an example of a case you have encountered?
- What is the role of [insert traditional healers or nurses] in treating people with mental illness?

*Questions for focus group discussion with both traditional healers AND nurses*

- How can traditional healers help patients with mental illness?
- How can nurses and doctors help patients with mental illness?
- Are there some types of patients with mental illness that should see a clinical officer or nurse? If so, at what stage(s)?
- Are there some types of patients with mental illness that should see a traditional healer? If so, at what stage(s)
- Can traditional healers and nurses work together to look after people with mental illness?
- How can they work together?
- Can they share clinical information, e.g. about early warning signs of relapse, side effects, suicidal risk?
- Can you foresee any problems of working together?

Second set of focus group discussions

*Questions for both separate and combined focus group discussions*

- How did the last focus group change your attitude towards collaboration with traditional healers/nurses?
- How did it change your practice? Did you liaise more with healers/nurses than you would have done before the focus group?
- Could some patients benefit from both hospital and traditional medicine for mental health problems? Can you foresee any problems of patients accessing both treatments at the same time?
- Could we establish a formal referral pathway? How would it work?
- Having a traditional medicine office at the hospital was suggested. How would it work? Who would be stationed at the office? Who would they be paid by? Would that person liaise with other healers? How can we be sure we are recommending good/trustworthy healers to patients?
- Would healers be interested in learning about mental health from nurses/doctors, or vice versa?
